# Quantification of ventilatory control in sleep apnea: from physiological insight to computable loop gain

**DOI:** 10.1093/sleep/zsag155

**Published:** 2026-06-18

**Authors:** Thijs Nassi, Eline Oppersma, Dirk W Donker, M Brandon Westover, Robert J Thomas

**Affiliations:** Beth Israel Deaconess Medical Center, Harvard Medical School, Boston, MA, United States; Cardiovascular and Respiratory Physiology, TechMed Center, University of Twente, Enschede, The Netherlands; Cardiovascular and Respiratory Physiology, TechMed Center, University of Twente, Enschede, The Netherlands; Cardiovascular and Respiratory Physiology, TechMed Center, University of Twente, Enschede, The Netherlands; Department of Intensive Care, University Medical Center Utrecht, Utrecht, The Netherlands; Beth Israel Deaconess Medical Center, Harvard Medical School, Boston, MA, United States; Beth Israel Deaconess Medical Center, Harvard Medical School, Boston, MA, United States

**Keywords:** obstructive sleep apnea, central sleep apnea, loop gain, ventilatory control instability, self-similarity, polysomnography, endotyping, precision medicine

## Abstract

Sleep-disordered breathing reflects the interplay of upper-airway collapsibility, sleep depth/arousability, and instability in ventilatory control. Ventilatory loop gain (LG) quantifies the latter as the ratio of ventilatory response to disturbance: values >1 indicate self-sustaining oscillations, whereas lower values denote stable control. Despite its clinical relevance, LG measurement remains largely confined to research laboratories because conventional protocols (e.g. controlled gas challenges, stepwise reductions in positive airway pressure) are invasive and technically demanding. Recent advances have enabled several computable LG estimation methods from signals available in routine polysomnography and selected home settings, creating a timely opportunity to translate LG beyond the laboratory. Indirect approaches include breath-hold maneuvers, cardiopulmonary-coupling metrics, respiratory self-similarity analysis, and data-driven or model-based estimation. Simplified surrogates improve accessibility but sacrifice physiological detail, whereas model-based methods (e.g. Phenotyping Using Polysomnography [PUP]) provide individualized LG profiles at higher requirements for signal quality and computation. Emerging evidence from model-based polysomnographic estimation indicates that a dynamic LG threshold near 0.7 may help identify patients who benefit from chemorespiratory stabilizers alongside obstruction-resolving therapies, whereas those with lower LG often respond adequately to anatomy-focused approaches alone; whether equivalent thresholds apply across estimation methods remains to be established. Prior reviews have emphasized physiology and phenotype-based care, but none have systematically compared LG assessment methods across a fidelity–feasibility spectrum or linked method choice to treatment selection and validation needs. This review synthesizes perturbation tests, signal-based surrogates, and model-based identification into a pragmatic framework with decision cues for screening versus confirmatory testing and priorities for clinical deployment.

## Introduction

Sleep apnea is a common, substantially underdiagnosed, and clinically consequential disorder—manifesting as daytime sleepiness, cognitive impairment, and increased cardiovascular risk [1–3]. Population estimates suggest that on the order of one billion adults worldwide have clinically relevant sleep-disordered breathing, underscoring the scale of the problem [4]. Effective management therefore hinges on accurate endotyping/phenotyping, as different underlying mechanisms require tailored therapeutic strategies [5, 6]. Among apnea driver mechanisms, ventilatory-control instability quantified by loop gain (LG), has emerged as a clinically useful and targetable trait [7–9]. LG captures the respiratory system’s response-to-disturbance ratio: higher LG implies a propensity for oscillatory, unstable breathing with frequent arousals, whereas lower LG reflects stable control [10, 11].

Historically, LG assessment relied on manual or semi-automated procedures (e.g. gas-perturbation tests, continuous positive airway pressure (CPAP) pressure drops [12–15]) that are physiologically direct but labor-intensive, expertise-dependent, and equipment-heavy, limiting use outside specialist labs. These methods advanced mechanistic understanding but have not scaled into routine care.

Over the past decade, advances in signal processing and mechanistic modeling alongside machine-learning approaches that leverage routine PSG signals have created a coherent pipeline from physiology, to algorithm, to clinical decision [16–18]. Because treatment response depends in part on whether ventilatory-control instability or anatomical collapse dominates, quantifying LG has direct therapeutic implications, a relationship detailed in the Therapeutic Implications section below.

Despite this progress, implementation barriers remain, best understood in order of clinical impact:

1. Validation and generalizability (multicenter evidence across comorbidities and demographics),
2. Signal quality and standardization (vendor differences, home vs. lab recordings),
3. Workflow integration (automation, reporting, and clinician interpretability), and
4. Contextual adaptation (e.g. altitude, heart failure, medication effects).

This review clarifies the physiological foundations, appraises state-of-the-art automated techniques, and delineates their clinical relevance and adoption pathways. We argue that modern computational approaches can deliver accurate, scalable, and practical LG quantification, and we outline a tiered path to translation, from automated PSG-derived screening to targeted confirmatory testing, designed to embed LG into routine phenotyping and treatment selection.

## Ventilatory control and LG

### Physiological concepts of ventilatory control

**Figure 1:**
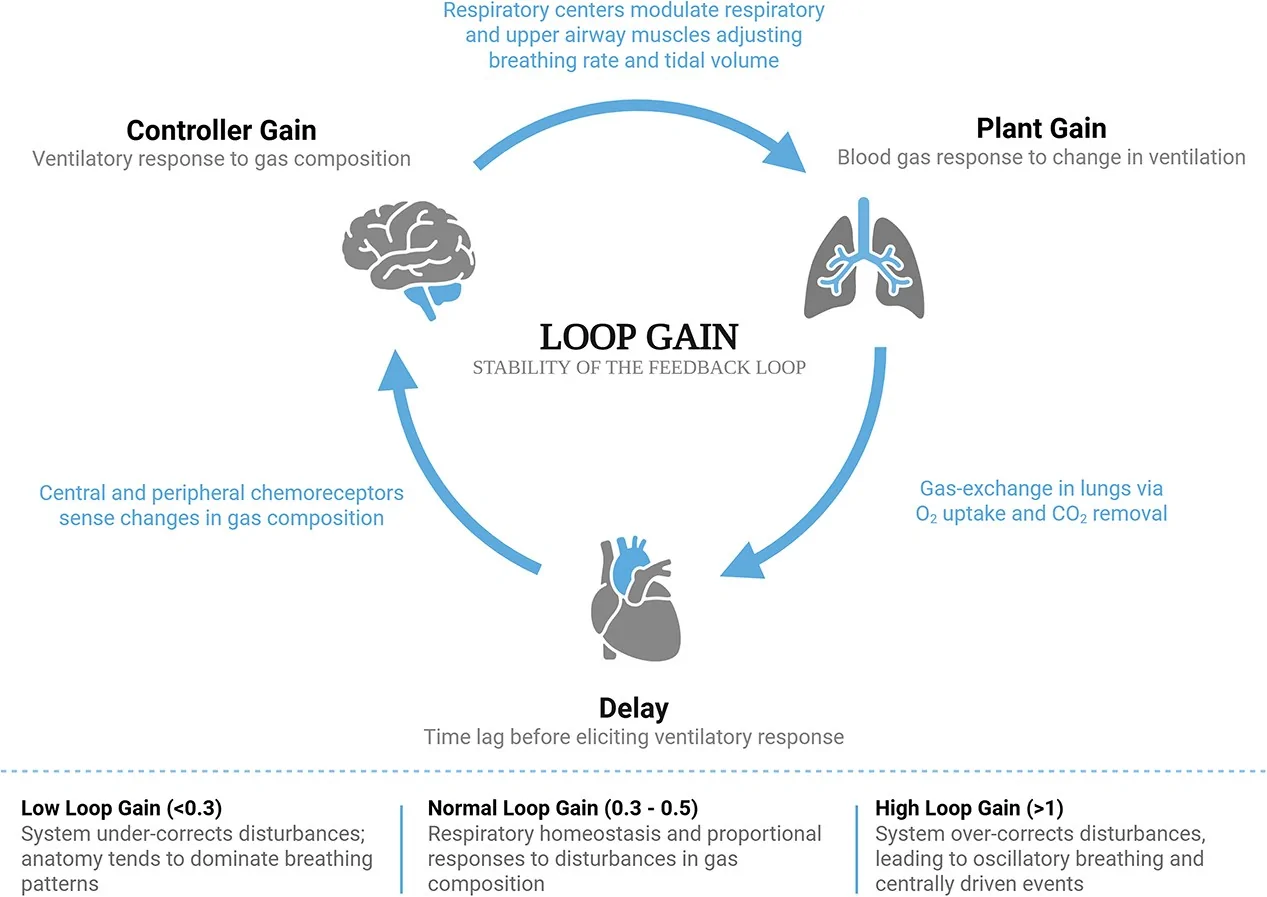
Schematic of the respiratory feedback loop illustrating controller gain (chemoreflex sensitivity of brainstem respiratory centers to changes in blood gasses), plant gain (how efficiently changes in ventilation alter blood gas composition via pulmonary gas exchange), and delay (circulatory and neural latency before a ventilatory response is expressed). Together, these components determine LG, the overall stability of the feedback loop. Low dynamic LG (<0.3) under-corrects disturbances and anatomy tends to dominate breathing patterns; normal dynamic LG (≈0.3–0.5) maintains respiratory homeostasis with proportional responses; and high dynamic LG (>1) over-corrects disturbances, predisposing to oscillatory breathing and centrally driven events. These ranges refer to LG evaluated at the system’s natural oscillation frequency, which accounts for both gain magnitude and feedback delay. Created in BioRender. Nassi, T. (2026) https://BioRender.com/824z5cy.

The ventilatory control system maintains respiratory homeostasis during sleep by responding primarily to changes in carbon dioxide (CO₂) levels, with additional input from O₂ sensing. Central chemoreceptors in the medullary retrotrapezoid nucleus express the proton-activated receptor GPR4 and respond to rising CO₂ by sensing the associated fall in local pH [19]. In contrast, peripheral chemoreceptors in the carotid and aortic bodies respond to low arterial O₂-levels, as well as to CO_2_ and pH changes [20]. These chemosensory signals are integrated by brainstem neural circuits, such as central pattern generators and afferent feedback loops, which modulate respiratory muscle activity to adjust both respiratory rate and tidal volume [21]. Mechanical properties of the respiratory system, including lung compliance and airway resistance, also influence how effectively these adjustments are carried out. LG, a concept adapted from control theory, quantifies the sensitivity of this feedback system [7, 8], as illustrated in Figure 1. It condenses the complex interplay of chemosensory, neural, and mechanical factors into a single, practical metric. While this simplification has limitations, LG is a valuable tool for identifying patients whose breathing instability stems from ventilatory control dysfunction, rather than anatomical obstruction [22]. Moreover, it clarifies the physiological overlap between obstructive sleep apnea (OSA) and central sleep apnea (CSA) [23].

### Understanding LG dynamics

LG describes how strongly the respiratory control loop reacts to a disturbance. When LG is well below 1, the system corrects only a fraction of each disturbance per breathing cycle. A LG of 0.5, for instance, restores half the perturbation per cycle, gradually returning to equilibrium, and upper-airway anatomy tends to dominate breathing patterns [9]. As LG approaches or exceeds 1, the correction matches or overshoots the original disturbance, and periodic breathing can become self-sustaining (Figure 1) [8, 24].

Whether the loop sustains oscillations, however, depends not only on the magnitude of the gain but also on the timing of the corrective response. The feedback delay, composed of circulatory transit time from lungs to chemoreceptors and neural processing latency, means that the ventilatory correction arrives after blood-gas conditions have already shifted [25]. When this delay places the correction sufficiently out of phase with the original disturbance, oscillations can emerge even when the static gain magnitude is below 1 [26]. The frequency at which this phase mismatch is most destabilizing defines the system’s natural oscillation frequency, typically corresponding to a cycle length of 25–60 seconds depending on circulation time. LG evaluated at this frequency, termed dynamic LG, captures magnitude and timing in a single value and is the clinically relevant stability metric; instability arises as dynamic LG approaches unity [8, 24].

Operationally, dynamic LG decomposes into three measurable components: the controller gain (chemoreflex sensitivity to CO₂ and O₂), the plant gain (how efficiently changes in ventilation alter PaCO₂), and the feedback delay [16, 22]. Higher controller gain or plant gain each amplify the magnitude of the loop’s response; longer delays shift its timing, causing corrections to arrive further out of phase. Because each component is independently shaped by physiology and pharmacology, dynamic LG can be raised or lowered through distinct mechanisms, a property that anchors the therapeutic strategies discussed below.

#### Clinical modulators

Factors that raise controller gain, increase plant sensitivity/narrow the CO₂ reserve, or prolong feedback delay elevate LG and promote periodic breathing (Figure 1); factors that blunt chemosensitivity lower LG and bias toward hypoventilation. For example, in heart failure, pulmonary congestion increases chemoreflex drive and prolonged circulation time adds delay, together narrowing the CO₂ reserve (distance from eupneic PaCO₂ to the apneic threshold) and predisposing to centrally driven events [27]. A similar high-gain state can arise when peripheral chemosensitivity is heightened and hypocapnia steepens the CO₂ response (e.g. high altitude) [28]. Conversely, opioids or neuromuscular weakness dampen chemosensitivity (low controller gain), tending toward hypoventilation rather than oscillation. These modulators frame the therapeutic choices outlined below. Finally, arousals interact with LG: a low arousal threshold causes frequent awakenings that interrupt the corrective ventilatory response before it can stabilize, while high-intensity arousals amplify the post-arousal ventilatory overshoot, increasing the expressed impact of elevated LG.

### Therapeutic implications

Because treatment response depends on which mechanism drives instability (ventilatory-control vs. anatomical collapsibility), measuring LG adds clinically actionable information for phenotyping and planning in sleep apnea [27]. In OSA, an elevated LG indicates that non-anatomical contributors, most commonly heightened chemosensitivity and/or prolonged feedback delay, can materially sustain periodic breathing and arousals [29]. In such patients, anatomy-only strategies (e.g. CPAP) may be insufficient, and adjunctive stabilization can be considered, such as supplemental O₂, carbonic anhydrase inhibition (e.g. acetazolamide) [30–35] or targeted CO_2_ reserve widening approaches [36–38]. Although a dynamic LG >1 denotes formal instability, clinical data derived from model-based polysomnographic estimation (PUP) suggest that values ≳0.7, as quantified by that method, represent a clinically relevant elevation that often warrants these adjuncts in addition to anatomy-directed care [16]. This threshold is method-specific; its transferability to other estimation approaches is discussed in the Assessment Methods section below.

In practical terms, each intervention targets a specific component of the feedback loop (Figure 1): acetazolamide and supplemental O₂ primarily lower controller gain. A small amount of controlled external dead space raises end-tidal CO₂ and widens the CO₂ reserve, lowering plant gain. Optimizing hemodynamics in heart failure can shorten the circulatory feedback delay. When LG is normal or low, CPAP, custom titratable mandibular advancement devices, or hypoglossal nerve stimulation, are appropriate and sedatives/opioids should be avoided or used cautiously [39, 40]. Targeting arousals via cognitive behavioral therapy for comorbid insomnia or via sedatives can also effectively reduce the expression of LG effects (this may be how zolpidem improves idiopathic CSA).

Quantified LG enables clinicians to disambiguate mechanism, stratify risk, and personalize treatment [40, 41]. Misclassification or incomplete diagnosis can lead to suboptimal outcomes; for example, using CPAP alone in a high-LG patient may fail to resolve symptoms and increase adverse outcomes [42, 43]. Integrating precise LG assessment into routine practice improves patient-centered outcomes by revealing whether instability is driven by chemosensitivity/feedback delay (high LG) or anatomical collapsibility (normal/low LG) [44, 45]. Therapy should then be aligned accordingly: adjunctive chemorespiratory stabilization for elevated LG, and anatomy-first strategies for normal/low LG.

Realizing these therapeutic benefits in practice requires reliable, scalable measurement of LG. The next section compares the estimation methods available, organized along a fidelity–feasibility spectrum ranging from direct physiological perturbations to automated analysis of routine clinical signals.

## Assessment methods for LG

Before comparing individual methods, an important caveat is warranted: absolute LG values are not directly comparable across approaches. Perturbation tests quantify gain at the specific frequency and operating point of an imposed disturbance. Model-based methods infer dynamic LG from spontaneous breathing, typically at the system’s natural frequency during sleep. Markers of expressed instability do not estimate LG directly, but instead reflect downstream consequences of control instability, such as periodic breathing morphology or breath–heart coupling. Because these methods rely on different assumptions about plant dynamics, arousal coupling, and upper-airway mechanics, numeric thresholds cannot be transferred across techniques. Establishing cross-method concordance and mapping decision boundaries between approaches is therefore essential for clinical translation (see Validation & Clinical Utility below).

**Figure 2:**
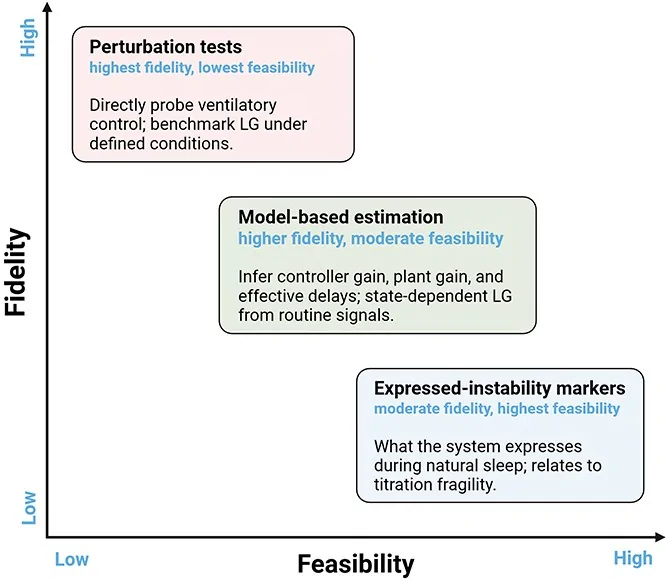
Conceptual placement of three families of ventilatory LG methods along a fidelity–feasibility spectrum. Perturbation tests (e.g. hypoxic–hypercapnic gas challenges, hyperoxic challenge, CPAP pressure drops, breath-holding) provide the highest physiological fidelity but lowest feasibility, directly probing ventilatory control under defined conditions. Model-based estimation from routine polysomnography offers higher fidelity with moderate feasibility, inferring controller gain, plant gain, and effective delays—and thus state-dependent LG—by fitting mechanistic feedback models to spontaneous breathing. Expressed-instability markers (e.g. cardiopulmonary coupling, SS, breath-by-breath probability of obstruction) have moderate fidelity but highest feasibility, capturing the instability that the system expresses during natural sleep and scaling to large cohorts and home monitoring. Created in BioRender. Nassi, T. (2026) https://BioRender.com/6izrh5g.

**Figure 3:**
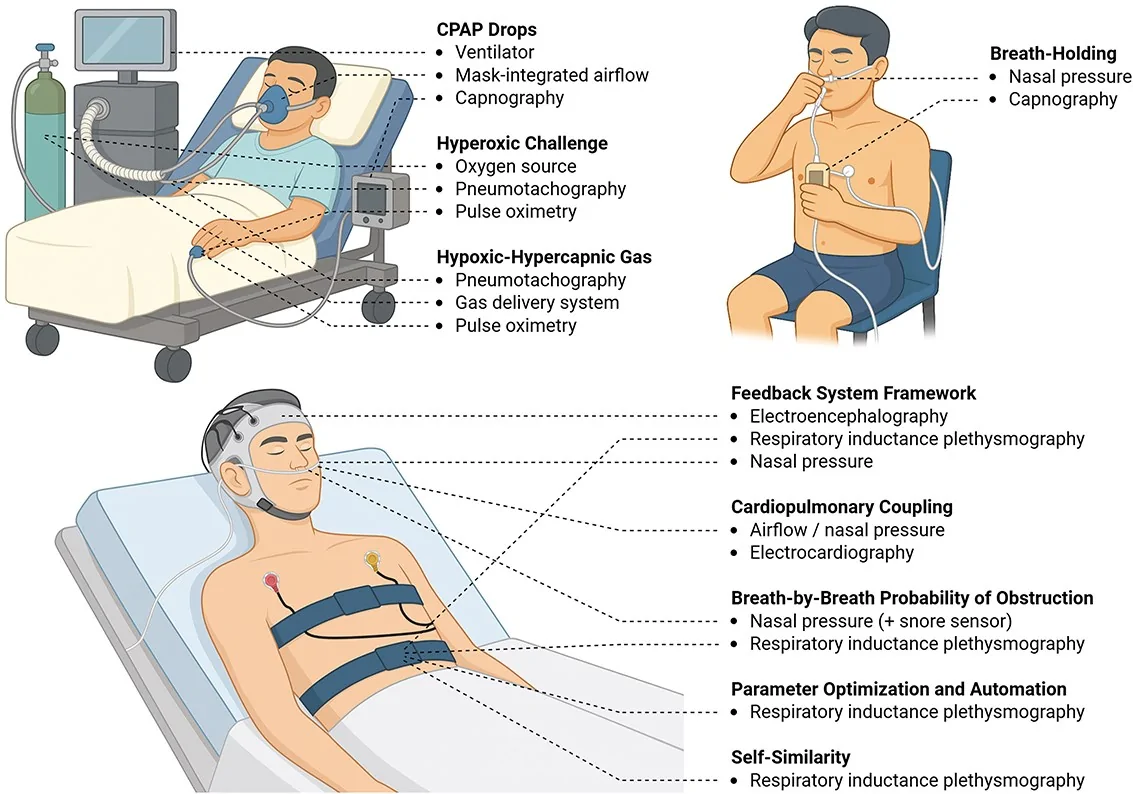
Signals and equipment required for representative LG assessment methods. Direct tests—CPAP pressure drops, hypoxic–hypercapnic gas challenge, and breath-hold maneuvers—require ventilator or gas-delivery systems plus monitoring (capnography, pneumotachography or nasal pressure, pulse oximetry). Signal-derived surrogates—mechanistic (feedback-system) modeling, CPC, SS, and automated parameter estimation—Primarily use routine PSG channels (RIP belts, nasal pressure/airflow, EEG/ECG). Dotted leaders indicate the primary sensors/hardware each method depends on. Created in BioRender. Nassi, T. (2026) https://BioRender.com/44tmnmp.

Estimation techniques for ventilatory LG can be organized along a fidelity–feasibility spectrum (Figure 2) and grouped into three families. We begin with direct physiological perturbations—hypoxic–hypercapnic gas challenges, hyperoxic challenge (Dejours test), and CPAP pressure-drop titrations, all of which anchor the high-fidelity end by causally probing the chemoreflex–plant loop; we also include breath-holding as a wake, open-loop maneuver with lower closed-loop relevance and fidelity but higher feasibility. As summarized in Figure 3, these require protocolized delivery systems (e.g. gas-mixing or CPAP hardware) and calibrated flow measurement. Next, expressed-instability markers, notably cardiopulmonary coupling (CPC), self-similarity (SS), and breath-by-breath probability of obstruction (P_obs_), leverage routine PSG channels and scale to lab and home settings; Figure 3 maps their minimal incremental setup, i.e. respiratory inductance plethysmography (RIP) belts, nasal pressure/airflow, and single-lead electrocardiogram (ECG) or peripheral photoplethysmography (PPG). Finally, model-based closed-loop identification from routine PSG (feedback-system modeling) occupies the middle ground: by fitting mechanistic dynamics to belts/airflow, it can recover LG and its components: controller gain, plant gain, and feedback delay, when identifiability conditions are met.

**Figure 4:**
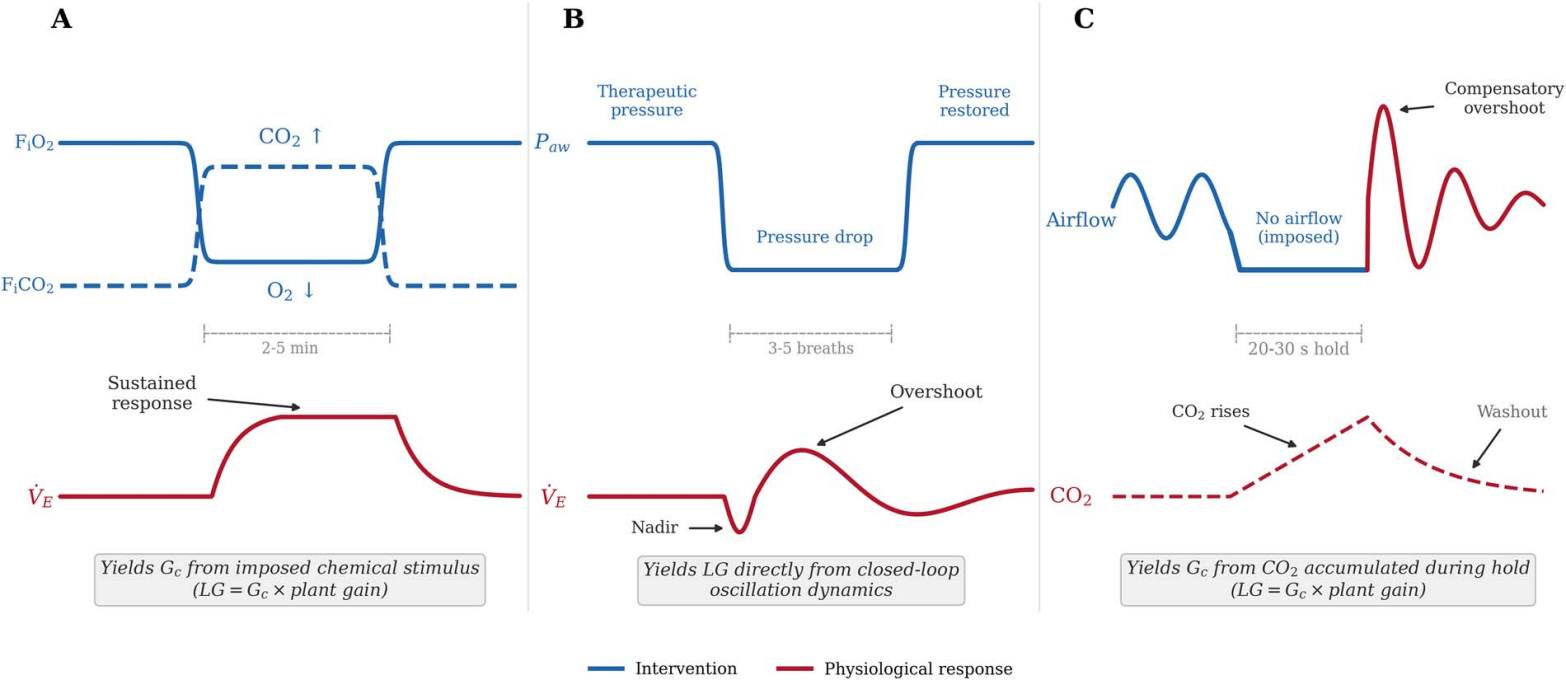
Schematic protocol timelines for representative perturbation tests. Solid and dashed lines distinguish primary and secondary signals. Gray boxes indicate the estimated parameter: controller gain (Gc, the chemoreflex sensitivity) or LG (the product of Gc, plant gain, and circulatory delay). (A) Hypoxic–hypercapnic gas challenge: controlled gas delivery (gas mixer, capnograph, pneumotachograph) with breath-by-breath targeting of end-tidal gasses; the ventilatory response (minute ventilation, V̇E) to the imposed change in inspired gas fractions (FiO₂, FiCO₂) yields Gc. (B) CPAP pressure-drop test: airway pressure (paw; CPAP device, flow sensor, mask pressure transducer) is reduced during stable NREM sleep; the overshoot amplitude and damping of the ventilatory oscillation yield LG directly. (C) Breath-hold maneuver (wakefulness): an end-expiratory hold (nasal pressure cannula, RIP belts, transcutaneous CO₂ monitor) followed by measurement of the compensatory ventilatory response and CO₂ washout; the ratio of ventilatory overshoot to accumulated CO₂ yields Gc.

**Figure 5:**
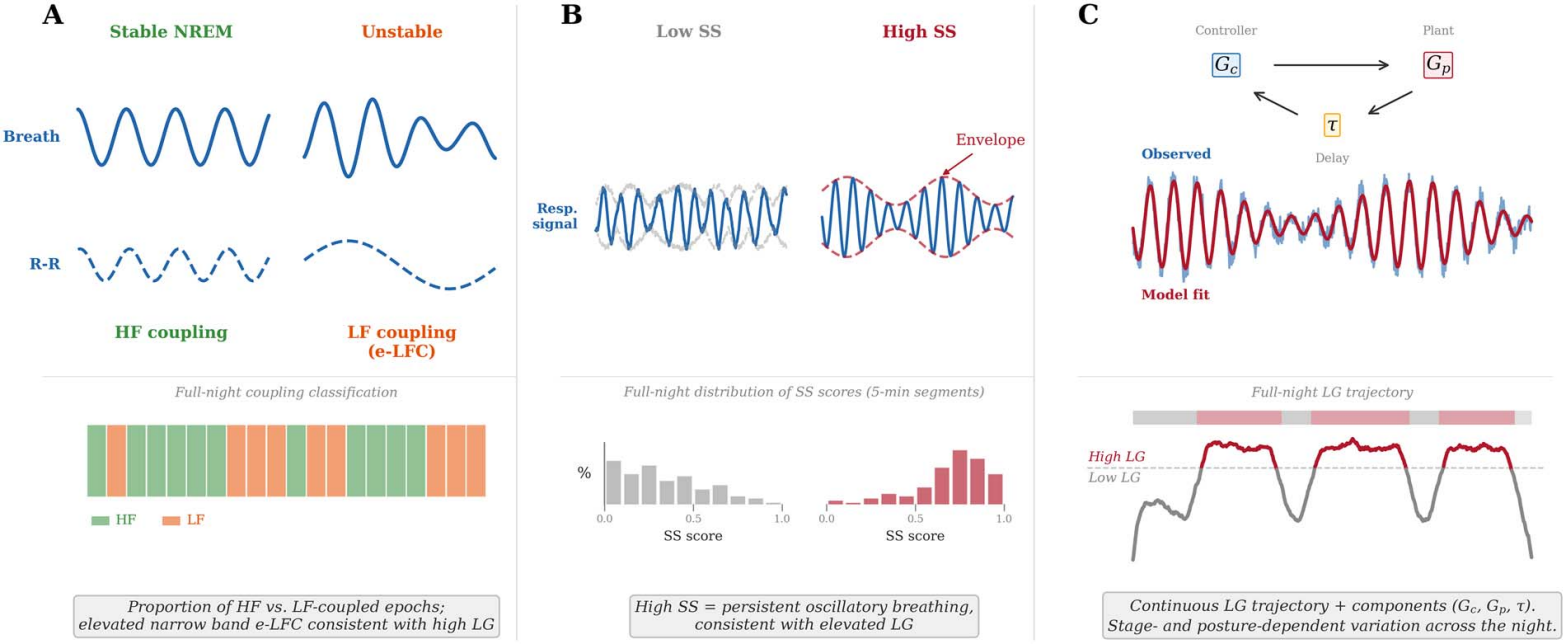
Computational pipelines for signal-derived and model-based LG estimation. These methods extract stability information from routine polysomnographic recordings. (A) CPC: cross-spectral coherence between cardiac rhythm (R-R intervals, dashed) and respiration (solid) classifies epochs as HF coupled (stable NREM, left) or LF coupled (narrow-band unstable breathing consistent with elevated LG, right). The full-night strip shows the resulting epoch-by-epoch classification. (B) SS: the amplitude envelope (dashed) of the respiratory signal is scored for periodic structure; irregular patterns yield low SS scores (left), while persistent self-similar waxing-waning yields high SS (right). Histograms show the distribution of per-segment SS scores across the night. (C) Model-based estimation: an explicit controller (Gc)–plant (Gp)–delay (τ) feedback model is fit to multi-channel PSG. The resulting continuous LG trajectory (lower panel) varies with sleep stage; segments exceeding the high-LG threshold are highlighted.

The remainder of this section follows that sequence (direct physiological perturbations, expressed-instability markers, model-based closed-loop identification) and, for each method, lays out the physiological principle; required signals and workflow; level of automation, yield, and feasibility; and key limitations. Table 1 provides a comparison of these characteristics across all methods. Figure 4 illustrates representative perturbation protocols. Figure 5 illustrates the computational pipelines underlying the expressed-instability markers and model-based estimation approaches.

**Table 1:** Overview of LG estimation methods and their characteristics

Method category	Method	Clinical setting	Equipment requirements	Event label requirements	Granularity	Evaluation	Validation status	Key advantages	Key limitations
Perturbation tests	Hypoxic-Hypercapnic Gas [12, 13, 46–48]	Research lab	Gas delivery system; pneumotachograph; capnograph; pulse oximeter	None	Event-by-event	Manual	Reference standard; cross-protocol concordance not established	Gold-standard chemoreceptor sensitivity	Expensive/time-consuming; disrupts sleep
	Hyperoxic challenge [50–55]	Research lab; In-lab PSG	Oxygen source; pneumotachograph; pulse oximeter	None	Event-by-event	Manual	Moderate; controller gain only; no head-to-head vs whole-loop LG	Trait-level controller-gain probe	Plant gain and delay not measured; protocols not standardized
	CPAP drops [14, 15, 56]	In-lab PSG	Ventilator; capnograph; mask-integrated airflow/pressure	Sleep staging	Event-by-event	Manual	Strong; validated vs gas challenges; benchmark for model-based methods	Direct plant-gain estimate	Expensive/time-consuming; disrupts sleep
	Breath-Holding [57–59]	Clinics	Capnograph; nasal pressure	None	Event-by-event	Manual	Limited; wake-only; correlated with PUP-LG; no clinical thresholds	Non-invasive; no special gear	Awake state where airway tone and drive differ
Expressed-instability markers	CPC [60, 61]	In-lab PSG	ECG; respiration sensor	Sleep staging	Continuous coupling index	Semi-Automated	Emerging; validated vs event indices; no direct LG concordance	Non-invasive; wearable-friendly; continuous	Indirect LG; sensitive to autonomic noise
	SS [62, 63]	In-lab PSG; home monitoring	RIP (or nasal pressure)	Sleep staging	5-minute segments	Automated	Emerging; predicts CPAP-emergent instability, AUC 0.82; no direct comparison to physiological LG	Non-invasive; wearable-friendly	Indirect LG; sensitive to artifacts
	Breath-by-Breath Probability of Obstruction 67	In-lab PSG	Nasal pressure + snore sensor; RIP	None	Breath-by-breath	Automated	Moderate; validated vs esophageal pressure; no treatment-response data	Non-invasive; uses routine signals; generalizes across cohorts	Indirect LG; sensitive to artifacts; ambiguous mid-range probabilities
Model-based estimation	Feedback System Framework [16, 17]	In-lab PSG	Nasal pressure	Sleep staging; Breathing events; EEG arousals	7-minute segments	Semi-Automated	Moderate–Strong; ICC >0.8 vs CPAP drops; needs multi-site replication	Non-invasive; Physiologically interpretable model	Computationally complex; Evaluation expertise
	Parameter optimization & Automation [18]	In-lab PSG	RIP (or nasal pressure)	Sleep staging	8-minute segments	Automated	Emerging; validated on synthetic data; expected phenotype associations confirmed; no head-to-head vs perturbation LG	Non-invasive; Mechanistic parameters; interpretable model	Computationally complex; Evaluation expertise

*ECG = electrocardiography; EEG = electroencephalograph.*

### Perturbation tests

#### Hypoxic-hypercapnic gas challenges

This test administers controlled gas mixtures with reduced O₂ and elevated CO₂ to activate chemoreceptors and elicit a ventilatory response. In the laboratory, end-tidal gasses are targeted breath-by-breath while ventilation is recorded, and CO₂ is tracked with capnography or transcutaneous monitoring [12, 13, 46–48] (Figure 4A). LG is estimated near eupnea as system gain (the proportional change in ventilation per unit imposed gas stimulus) with feedback delay accounted for. Many protocols primarily quantify controller gain, with closed-loop LG obtained when plant and delay are modeled explicitly.

This approach offers high physiological fidelity (Figure 2) but requires calibrated gas-delivery hardware (Figure 3), trained staff, continuous safety monitoring, and strict protocol adherence, which limits use outside specialized laboratories (Table 1).

Gas challenges serve as the physiological reference standard for chemoreflex sensitivity and, when plant and delay are modeled, for closed-loop LG [12, 13]. However, protocols vary (single-breath CO₂, pseudorandom binary stimulation, rebreathing) and cross-protocol concordance has not been formally established. Standardized protocols are needed so that gas challenges can serve as a consistent benchmark for validating automated methods [49].

#### Hyperoxic challenge

This maneuver, also referred to as the “Dejours test”, briefly substitutes room air with 100% oxygen to acutely silence tonic carotid-body input and observe the resulting fall in ventilation. In its classical form, ventilation (or respiratory rate and tidal volume) is measured during steady-state normoxia, then during a short hyperoxic pulse (typically 20–60 s); the magnitude and timing of the transient ventilatory depression over the next few breaths index the prevailing peripheral chemosensory drive [50]. Conceptually, the test probes the controller arm of the loop: by abruptly removing hypoxic afferent drive while leaving plant mechanics and circulation unchanged, it reveals how strongly ventilation depends on carotid-body activity at that operating point [50]. In experimental preparations, the ventilatory decline correlates closely with reductions in carotid sinus nerve discharge during the hyperoxic phase, supporting its interpretation as a measure of tonic chemoreflex drive and reflecting underlying hypoxia-sensing pathways, including H₂S-dependent mechanisms [50, 51]. In humans, variations of the Dejours test have been used to quantify carotid-body sensitivity in health and exercise [52], altitude acclimatization [53], and metabolic dysregulation (prediabetes) [54]; in OSA cohorts, the percent fall in respiratory rate after a brief O₂ pulse (e.g. >12% decrease) is taken as a marker of “high” carotid-body chemosensitivity and associates with higher apnea–hypopnea index (AHI), longer apneas, and greater rapid eye movement (REM)-related event burden, while simple PSG-based scores have been proposed to screen for this high-chemosensitivity phenotype [55]. In the fidelity–feasibility map (Figure 2), hyperoxic challenge offers high specificity for peripheral chemoreflex control (controller gain near eupnea) with relatively modest infrastructure compared to full hypoxic–hypercapnic titrations (oxygen source and fast-switching valve rather than full gas-mixing systems), making it attractive as a trait-level probe of loop gain; however, it provides only a partial view of the loop (plant gain and delay must be inferred or assumed), is sensitive to behavioral state and baseline PaCO₂, and lacks standardized cut-offs and protocols for routine clinical deployment (Table 1).

The Dejours test has been validated against carotid sinus nerve discharge in animal preparations [50], and in humans the percent ventilatory decline correlates with clinical markers of chemosensitivity [55]. However, it probes only peripheral controller gain, not whole-loop dynamic LG; no study has directly compared Dejours-derived sensitivity with closed-loop LG from CPAP drops or model-based estimation. Standardized cut-offs and prospective validation against treatment outcomes are still needed [49].

#### Continuous positive airway pressure drops

This perturbation changes airway pressure rather than inspired gas composition [14, 56]. In a CPAP-drop test, mask pressure is lowered by a small step for several breaths during stable non-rapid eye movement (NREM) sleep, then returned to baseline. This evokes a predictable ventilatory response that is recorded with mask-pressure and airflow sensors [15] (Figure 4B). The disturbance–response sequence is fit to a feedback model linking the pressure step to the change in minute ventilation (V̇E), with feedback delay included. A larger, faster overshoot indicates higher LG. A well-damped response indicates lower LG.

Compared with gas challenges, CPAP drops avoid gas-delivery systems and blood-gas sampling and leave inspired gasses unchanged, simplifying safety oversight and enabling use with standard PSG/clinical CPAP. However, limitations remain: the test requires precise pressure-control and leak management, upper-airway mechanics and lung-volume shifts can bias controller estimates, and patients with mask intolerance may be unsuitable. Automated pipelines reduce manual work but need device-specific validation. Overall, CPAP drops trade a slight loss of physiological fidelity for greater practical feasibility (Figure 2; Table 1).

CPAP-drop LG has been validated against gas-challenge-derived chemical control stability [14] and serves as the physiological benchmark for model-based estimation methods [16, 56]. Within-lab reproducibility has been demonstrated, but multi-site concordance data and standardized protocols across CPAP devices remain limited [49].

#### Breath-holding techniques

Within the perturbation spectrum, breath-holding provides the most feasible option. It offers lower physiological fidelity than gas challenges or CPAP drops but requires minimal infrastructure and is easy to repeat. During wakefulness, end-expiratory breath-holds of fixed duration create a controlled rise in CO₂ and a fall in O₂, which elicit a compensatory ventilatory response at release. Ventilation is measured with nasal pressure or RIP, and CO₂ is tracked with end-tidal or transcutaneous sensors. LG is then approximated with a gain-type metric: the proportional change in V̇E per unit rise in CO₂ above eupnea, calculated around the resting level. Delay and damping are captured by the time to peak response and the recovery time constant. Multiple trials are averaged to improve reliability [57–59] (Figure 4C).

Interpretation requires context. Wakefulness preserves pharyngeal tone and allows volitional control, so results primarily index chemosensitivity and delay rather than upper-airway behavior during sleep. Standardization of lung volume, posture, coaching, hold duration, and leak control is essential, and autonomic conditions or medications can alter the accompanying heart–breath dynamics. In practice, breath-holding provides a minimal-infrastructure, same-visit assessment (no overnight stay) useful for low-cost serial monitoring when coarse accuracy is acceptable. Formal sleep-state LG is better measured with CPAP drops or gas challenges (Table 1).

Messineo et al. demonstrated a correlation between breath-hold-derived chemosensitivity and PUP-estimated LG in patients with OSA [59]. However, the maneuver is performed during wakefulness and has not been validated against sleep-state LG from perturbation tests. No standardized protocol or clinical threshold has been established [49].

### Expressed-instability markers

#### Cardiopulmonary coupling

CPC characterizes how ventilation and cardiac rhythm couple and co-vary across the night. From a single-lead ECG or pulse plethysmography (PPG) signal, a respiratory rate (RR) interval series and an ECG-derived respiration (EDR) or PPG-derived respiration (PDR) signal can be obtained (or RR can be paired with nasal pressure or abdominal/thoracic effort recordings) and their cross-spectral coherence and phase computed [60, 61]. During stable NREM sleep, vagal tone produces respiratory sinus arrhythmia—heart rate rises with inspiration and falls with expiration. In CPC, this tight breath–heart synchrony appears as high-frequency (HF) coupling and reflects stable ventilation and stable NREM sleep. When ventilatory control becomes unstable, consistent with higher effective LG, ventilation waxes and wanes in slow cycles of about 30–90 seconds. These slow oscillations entrain autonomic outflow, shifting the breath–heart linkage into the low-frequency (LF) band (Figure 5A). The CPC trace then shows LF-dominant epochs, a characteristic sign of unstable breathing, and high power of LFC (elevated LFC, e-LFC) generally aligning with clinical apneas and hypopneas. If these oscillations are metronomic, they are likely driven by carotid body-based respiratory chemoreflexes and have a “narrow band” pattern (e-LFC_NB_). CPC is typically estimated in sliding windows and summarized as the proportion of HF- versus LF (including e-LFC_NB_)-dominant coupling over the night, offering a scalable, non-invasive indicator of ventilatory-control stability that can be obtained from ECG/PPG alone (including wearables). Interpretation remains contextual: autonomic conditions or drugs, arrhythmias/pacing, sleep-stage transitions, and signal quality (noisy EDR/PDR, mouth-breathing if nasal pressure is used) may blur the respiratory link and should temper inference (Table 1).

CPC has been validated against manually scored respiratory event indices and associated with adaptive servo-ventilation treatment efficacy [60, 61]. As an expressed-instability marker rather than a direct LG estimate, CPC requires concordance studies to establish its quantitative relationship with dynamic LG and method-specific clinical thresholds for treatment decisions [49].

#### Self-similarity

Within the expressed-instability marker family, SS sits toward the scalable end (Figure 2) because it requires only a respiratory signal and no cardiac, gas, or pressure measurements. In contrast to CPC, which quantifies breath–heart coherence, and mechanistic modeling, which estimates control parameters, SS evaluates how strongly the respiratory pattern repeats over time using RIP or nasal-pressure envelopes. The algorithm applies sliding-window autocorrelation and template matching to the envelope and emphasizes slow-cycle structure; persistent waxing–and-waning produces high SS and is consistent with oscillation-prone control (an association, not a direct LG estimate). Irregular timing, fragmented events, or frequent arousals lower SS and may suggest alternative drivers of instability that are less dominated by chemoreflex gain (Figure 5B) [62, 63].

SS is fully automated, non-invasive, and scalable, and has been clinically validated against CPAP-emergent respiratory instability [62, 63]. However, it was not developed through direct comparison with a physiological gold-standard LG measure [64]. Current evidence is based on small observational studies; key next steps include prospective validation in independent populations, direct comparison with perturbation-derived LG, and definition of clinical thresholds and a minimal clinically important difference [49]. Nonetheless, high respiratory SS during sleep appears to have adverse biological consequences [65, 66].

#### Breath-by-breath probability of obstruction

Using routine PSG channels (airflow, thoracoabdominal effort, snore) an automated classifier yields a per-breath probability of obstruction (P_obs_), which places each breath on an obstructive–central continuum [67]. Feature patterns of flow flattening, paradoxical effort, snore timing, and desaturation kinetics are mapped to P_obs_; higher values indicate greater inferred upper-airway resistance, lower values favor reduced drive. As a surrogate measure, P_obs_ is validated (not trained) against esophageal pressure–normalized resistance on a breathwise basis, correlates with subject-level obstructive burden, generalizes across cohorts, and shows high night-to-night reproducibility—placing it at the high-feasibility end among the expressed-instability marker options. Rather than estimating LG directly, it summarizes the expressed event phenotype that often co-varies with control instability. Ambiguous mid-range probabilities are expected in mixed physiology, and performance can degrade with artifacts, leaks, or device heterogeneity (Table 1). Because Pobs indexes event phenotype rather than oscillatory instability, it is not included in Figure 5. Remaining validation priorities include prospective studies linking Pobs-classified event phenotype to endophenotype-guided treatment response and establishing thresholds that would trigger confirmatory LG testing [49].

Collectively, these expressed-instability markers, CPC, SS, and Pobs, are well suited for screening/triage and longitudinal trending, with higher-fidelity testing reserved when precise sleep-state LG is decision-critical (Figure 2).

### Model-based estimation

#### Feedback system framework

Model-based closed-loop identification sits between direct perturbations and the expressed-instability markers on the fidelity–feasibility spectrum (Figure 2). Rather than classifying signal patterns, it posits an explicit feedback system for ventilatory control and estimates its parameters from routine PSG. Simplified Mackey–Glass–style or linear controller–plant models with feedback delay reproduce spontaneous sleep breathing. In this formulation, ventilatory drive combines a chemical drive (chemosensitivity to PaCO₂/PaO₂ sensed centrally and peripherally) with an arousal-related surge after cortical arousals; the plant maps changes in alveolar ventilation to PaCO₂ with characteristic time constants and a delay. After fitting, LG is evaluated near the patient’s operating point (typically at the system’s natural frequency) and reported with delay, giving a physiologically interpretable index of stability [16, 17].

Because these methods deliver parameter-level insight (controller gain, CO₂ reserve/plant behavior, delay, damping) from standard signals, they offer greater insight into a patient’s ventilatory control physiology than the expressed-instability markers. Assessment of LG on spontaneous respiratory events rather than actively perturbing the control system prioritizes ecological validity and scalability from routine PSG and preserves native sleep state. However, estimates become more model-dependent, vulnerable to arousal-coupled drive and variable obstruction/leaks, and therefore carry wider uncertainty compared to CPAP drops or gas challenges. The direct perturbation techniques yield a higher signal-to-noise ratio and their causal identification with tighter confidence intervals at the expense of specialized equipment, protocol time, and potential sleep disruption.

#### Parameter optimization and automation

Building on this framework, modern pipelines treat the drive to breathe as a latent state estimated from overnight PSG. After preprocessing to automatically remove signal artifacts, the algorithm updates the latent state and patient-specific parameters (e.g. controller gain, delay, time constants). Using state-space/Bayesian filters with expectation–maximization (EM), it yields a continuous, stage-aware profile of LG and its underlying controller gain and delay. Importantly, this dynamic trajectory—rather than a single summary value—reveals within-night variation in ventilatory control. It can show stage-, posture-, and arousal-linked surges in LG, identify sustained high-LG windows where targeted intervention or confirmatory testing is most informative, and support longitudinal comparisons before and after therapy (titration studies; Figure 5C) [18].

Important limits remain. Separating controller from plant effects can be difficult. Variability of LG estimates that shift across sleep stages can be noisy and hard to interpret. Compute needs are modest, but credibility depends on verification, validation, and uncertainty quantification (VVUQ), including comparison to direct perturbation tests and routine uncertainty reporting so that outputs are interpretable and safe for clinical use (Table 1).

PUP-based LG shows good concordance with CPAP-drop-derived LG (intraclass correlation coefficient > 0.8) [16] and the ≳0.7 threshold has been associated with treatment response in early cohorts [16, 39]. Scalability was demonstrated in an independent Icelandic cohort [17]. Bayesian state-space approaches have been validated on synthetic data with known parameters and reproduce expected phenotype associations (e.g. higher LG in central apnea, heart failure, altitude, and NREM sleep), but still require independent replication and direct comparison with perturbation benchmarks. For all model-based methods, multi-site validation across diverse populations, device vendors, and comorbidity profiles, as outlined in the ATS endophenotyping research statement [49] (Table 1), remains the key barrier to clinical adoption.

With LG estimation methods now maturing, the challenge shifts from how to measure LG, to how to embed that measurement into clinical workflows and generate the evidence base needed for adoption.

## Integrating automated loop gain assessment into clinical practice: barriers and implementation strategies

Despite recent advances, translating automated LG into routine care requires coordinated progress on fronts not addressed by methodological development alone: embedding LG software into clinical platforms, educating clinicians beyond the AHI paradigm, managing signal quality across diverse settings, accounting for clinical modulators in interpretation, and generating the validation evidence needed for adoption. The remainder of this section addresses each in turn.

Clinical deployment depends on platforms that interface seamlessly with existing polysomnography (PSG) systems and reporting pipelines. Cloud-based tools (e.g. PUPpy) can enable point-of-care use with minimal engineering support when they provide robust device adapters, secure data handling, and clinician-facing summaries rather than engineering diagnostics [17].

Many sleep programs remain entirely anchored to the AHI; targeted education is required to normalize parameter-level metrics (LG, controller/plant gain, delay). Standardized training for physicians and technologists, paired with interdisciplinary case reviews, will accelerate correct interpretation and use in decisions (screening, titration, therapy selection).

Automated LG depends on reliable respiratory signals. The per-method signal requirements described above should inform standardized acquisition protocols; automated artifact detection and device-agnostic preprocessing are needed to handle the recording heterogeneity common in routine clinical PSGs.

Interpretation must account for the clinical modulators described earlier. Pharmacologic agents, cardiopulmonary disease, acid–base shifts, and supplemental gas therapy (see Clinical Modulators) can each shift the LG estimate. Automated reports should flag detected modifiers and, when appropriate, widen uncertainty intervals rather than apply over-precise point corrections.

### Validation and clinical utility

A recent ATS research statement on endophenotyping translation proposes a structured framework requiring each metric to demonstrate clinical relevance (predicting outcomes and treatment response), detectability (reliable, reproducible measurement in clinical settings), and treatability (modifiable by intervention with established minimal clinically important differences), and progress through derivation, external validation, and impact analysis before clinical adoption [49]. No endophenotypic metric, including LG, has yet fulfilled all criteria. The validation status described above, and the criteria below, follows this roadmap and delineates the remaining requirements for each class of LG estimation methods.

- *Analytical validity:* concordance with perturbation tests (gas challenges/CPAP drops), bias and limits of agreement, and sleep stage-specific performance.
- *Reliability and uncertainty:* test–retest stability across nights, explicit uncertainty quantification (per-window confidence/credible intervals), and transparent failure rates (e.g. unusable-signal proportion).
- *Predictive validity:* prospective ability to predict response or non-response to CPAP, supplemental O₂/acetazolamide, mandibular advancement, or hypoglossal nerve stimulation; decision-curve analyses to quantify net benefit.
- *Clinical utility and operations:* impact on time-to-effective therapy, avoided failed titrations, reduction in return sleep studies, and cost/process outcomes (setup time, technologist effort).
- *Generalizability and equity:* performance across age, BMI, sex, ancestry, comorbidity, device vendors, and centers; strategies for out-of-distribution detection and model recalibration.
- Trial blueprint: multi-site, prospective studies with pre-registered endpoints and comparators against standard-of-care AHI-guided management, including superiority or non-inferiority designs and patient-centered outcomes. Early deployment in research-oriented centers can enable controlled workflows and prospective evidence generation [45, 62], with pilots pre-specifying endpoints for predictive accuracy, treatment selection, and clinical outcomes.

One concern that spans all validation criteria is the night-to-night variability of LG estimates, which may limit the ability of a single measurement to accurately reflect the underlying endotype. That a sleep-breathing respiratory control endotype would be rigid is not realistic, as there are dynamic modulators such as body position, sleep state, sleep depth and time of night, which will impact any computation. Moreover, an “average” for one night necessarily will include stable breathing periods and REM sleep, where expressed high LG is rare. Multi-night estimates will provide greater confidence of the true LG value, but ultimately any metric will have to be considered together with features such as REM- vs. NREM-dominant, oximetry patterns, arousal positioning, airflow recovery patterns, and persistence or induction of instability by anti-obstructive therapies.

Looking ahead, priorities include AI-assisted real-time feedback, wearable integrations for longitudinal tracking, and multi-site VVUQ that anchors automated LG to causal perturbation benchmarks while reporting uncertainty in a clinician-legible way. As these elements mature, adoption should shift sleep-apnea management toward more personalized, physiology-informed care.

## Conclusion

Unlocking the full potential of automated LG assessment calls for coordinated progress on four fronts: practical software solutions, clinician education, robust technical refinement, and multicenter validation. Meeting these requirements will embed LG estimation seamlessly into PSG workflows and clinical decision systems. Once readily available, LG offers a scalable phenotyping metric that directs patient-specific combinations of anatomical and ventilatory-control therapies for sleep apnea. Implemented in this way, automated LG assessment can move precision respiratory care from research settings into routine clinical practice.
